# Supplementary material for: Seminoma and Embryonal Carcinoma Footprints Identified by Analysis of Integrated Genome-Wide Epigenetic and Expression Profiles of Germ Cell Cancer Cell Lines
Source: PLoS One. 2014 Jun 2;9(6):e98330. doi: 10.1371/journal.pone.0098330 (PMC4041891; doi:10.1371/journal.pone.0098330)
Supplement: File S1 — ZIP file containing DMRforPairs output for significant regions. Please start from the html files. (ZIP) [file pone.0098330.s008.zip › figures/6101.pdf]

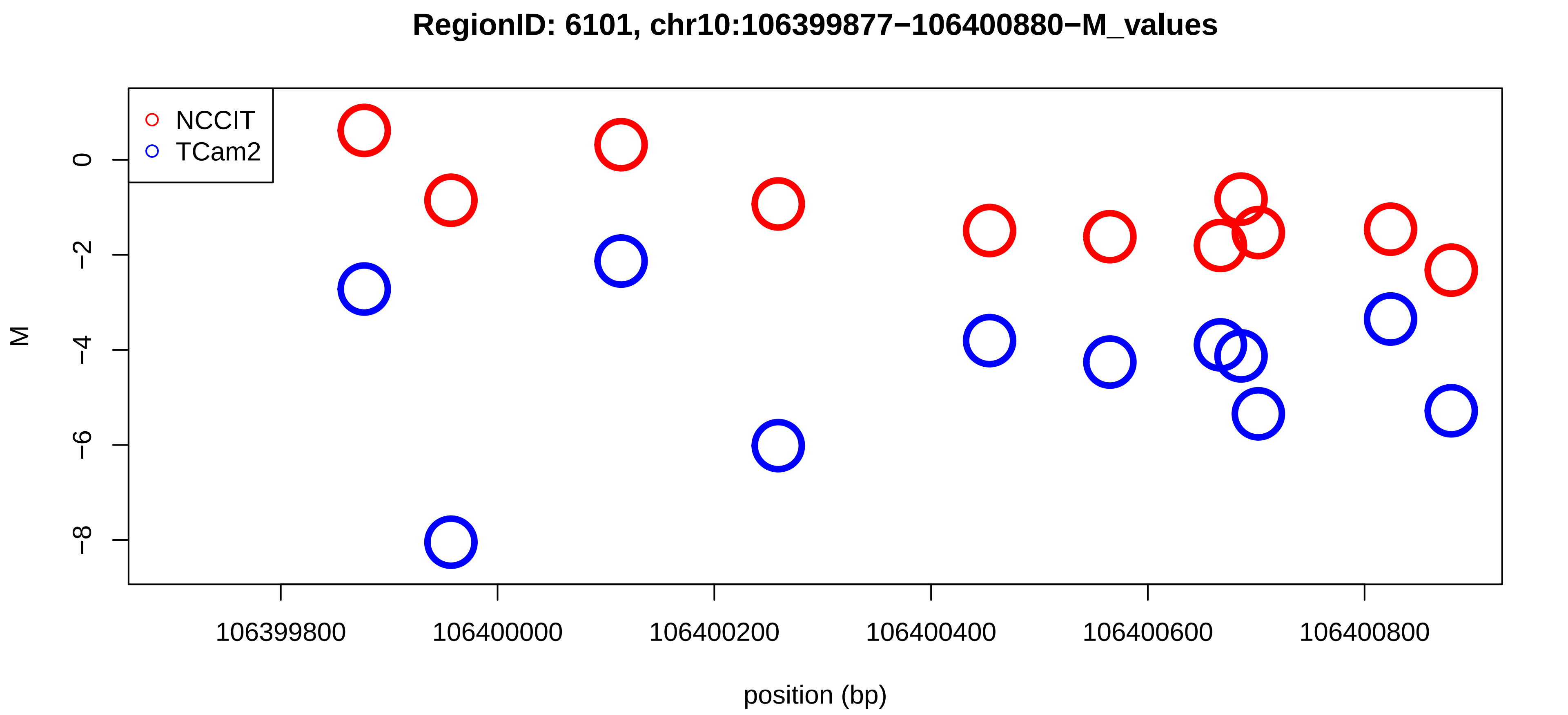
RegionID: 6101, chr10:106399877−106400880−M_values
NCCIT
TCam2
M
0
−2
−4
−6
−8
106399800
106400000
106400200
106400400
106400600
106400800
position (bp)

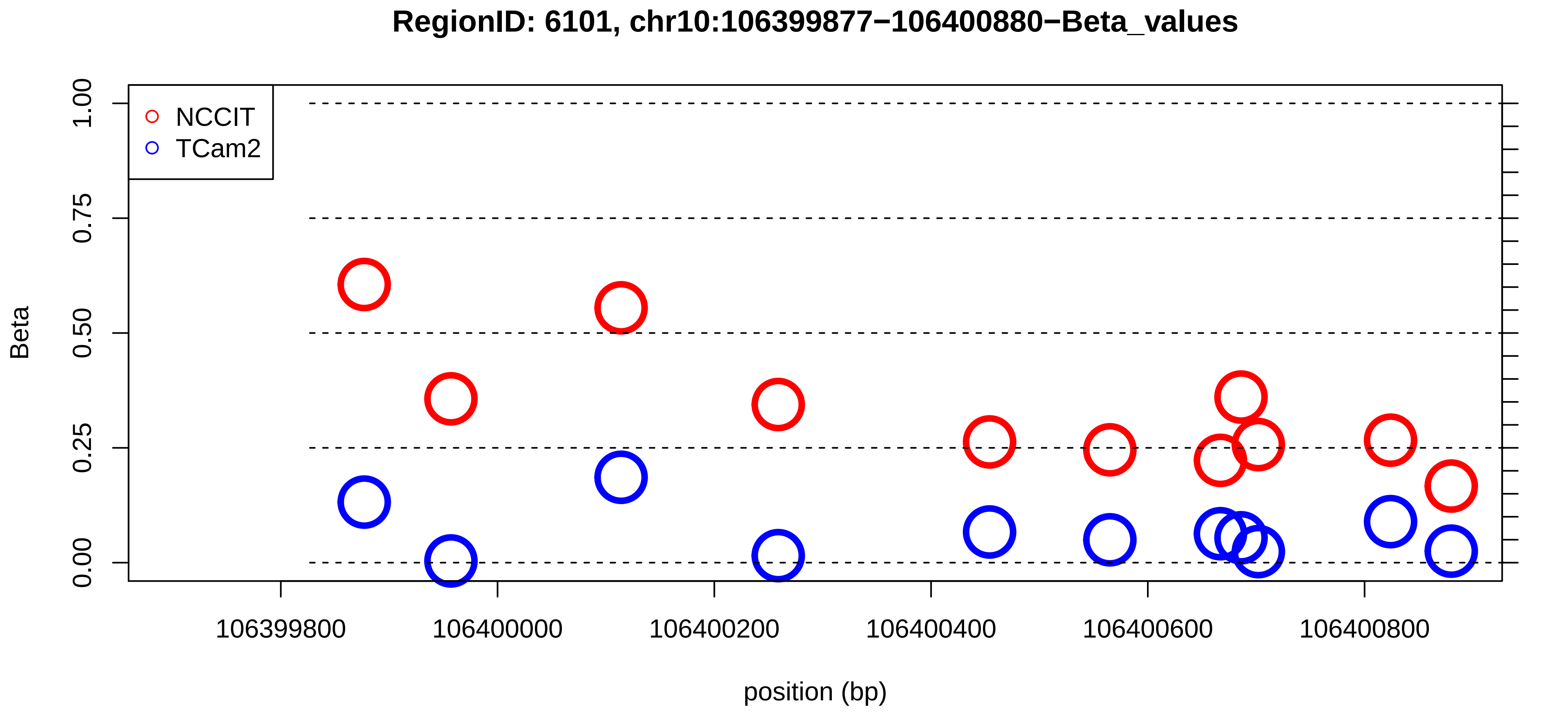
RegionID: 6101, chr10:106399877−106400880−Beta_values
NCCIT
TCam2
Beta
1.00
0.75
0.50
0.25
0.00
106399800
106400000
106400200
106400400
106400600
106400800
position (bp)

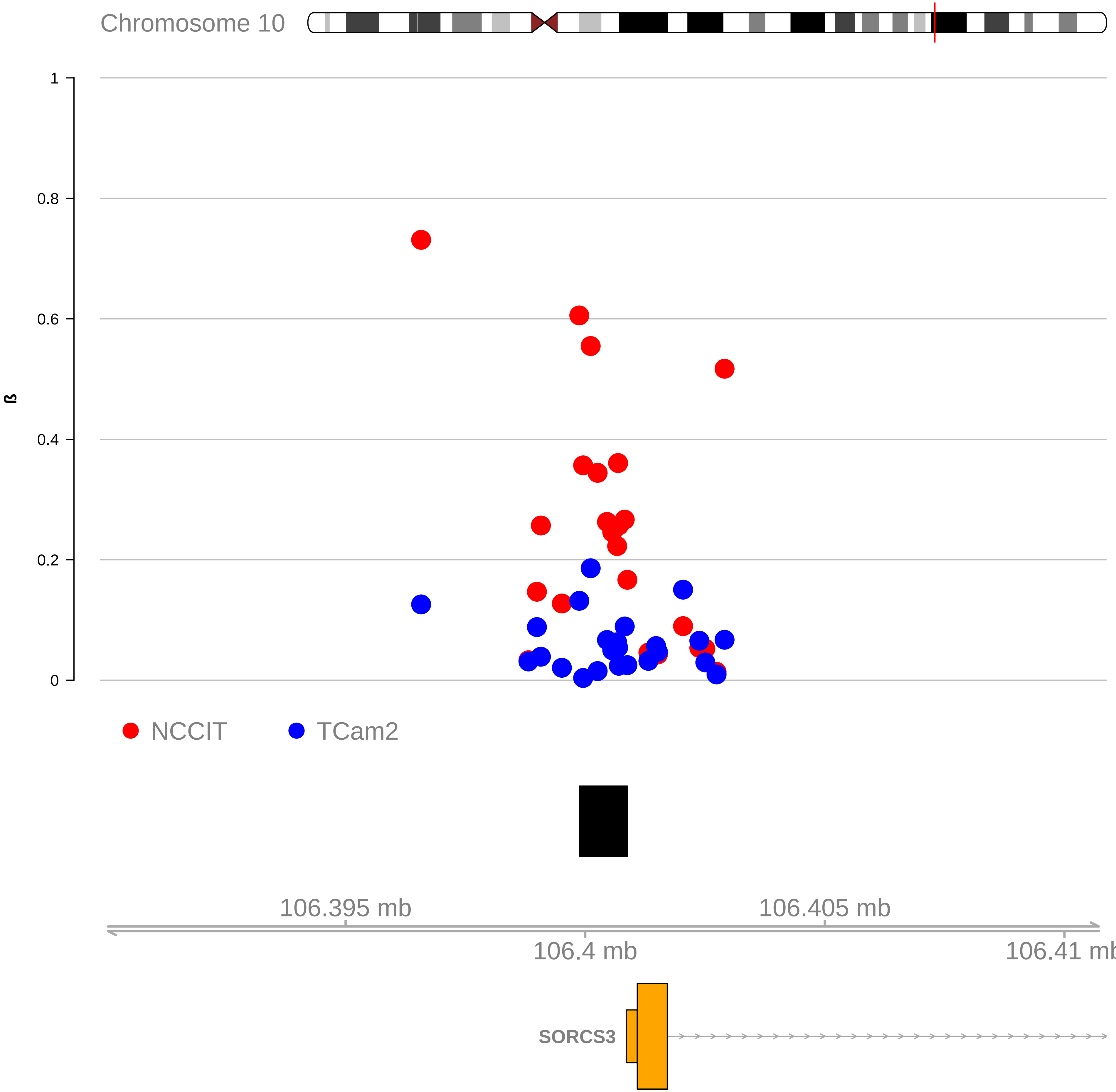

Chromosome 10
1
0.8
0.6
0.4
0.2
0
β
NCCIT
TCam2
106.395 mb
106.405 mb
106.4 mb
106.41 mb
SORCS3
